# MolProphecy: Bridging medicinal chemists’ knowledge and molecular pre-trained models via a multi-modal framework^☆^

**DOI:** 10.1016/j.jare.2025.10.035

**Published:** 2025-10-25

**Authors:** Jianping Zhao, Qiong Zhou, Tian Wang, Yusi Fan, Qian Yang, Li Jiao, Chang Liu, Zhehao Guo, Qi Lu, Fengfeng Zhou, Ruochi Zhang

**Affiliations:** a College of Computer Science and Technology, Changchun University of Science and Technology, Changchun, Jilin 130012, China; b College of Computer Science and Technology, Jilin University, Changchun, Jilin 130012, China; c Key Laboratory of Symbolic Computation and Knowledge Engineering, Ministry of Education, Jilin University, Changchun, Jilin 130012, China; d Department of Chemical and Biological Engineering, The Hong Kong University of Science and Technology, Clear Water Bay, 999077, Hong Kong, China; e Beijing Life Science Academy, Beijing, 102209, China; f Communication University of China, Beijing, 102209, China

**Keywords:** Molecular property prediction, Large language models, Proxy-human-in-the-loop, Multi-modal fusion, Interpretability, Drug discovery

## Abstract

****Introduction**:**

In drug discovery, the tacit domain knowledge of experts plays a critical role in guiding molecular design and decision-making.However, existing molecular pre-trained models rarely incorporate such expert knowledge, leading to suboptimal molecular design decisions.

****Objectives**:**

This study proposes MolProphecy, a proxy-human-in-the-loop (proxy-HITL) multi-modal framework that integrates chemists’ domain knowledge with structural molecular information to improve predictive accuracy and interpretability in drug discovery.

****Methods**:**

MolProphecy simulates chemist reasoning using ChatGPT to generate expert-level chemist insights for target molecules.This knowledge is encoded by a large language model (LLM) and fused with graph-based molecular features via a gated multi-head cross-attention module.The framework jointly reasons over human-derived and structural information.

****Results**:**

MolProphecy consistently outperforms baseline models across nine MoleculeNet benchmarks.For illustration, on FreeSolv it achieves an RMSE of 0.796, a 9.1% reduction over the best baseline.On BACE, SIDER, and ClinTox, MolProphecy improves AUROC by 5.39%, 1.43%, and 1.06%, respectively.In addition, MolProphecy demonstrates strong generalization on an independent solubility dataset, demonstrating robustness and applicability of our multi-modal fusion framework.

****Conclusion**:**

MolProphecy provides a generalizable framework for molecular property prediction by integrating simulated chemist expertise with structural data. Its design allows simulated input to be replaced with real chemist knowledge without retraining, establishing a pathway toward collaborative and interpretable drug discovery.

## Highlights

• Proxy-human-in-the-loop (HITL) integration.MolProphecy integrates chemist expertise as an independent knowledge modality:currently simulated via ChatGPT, yet seamlessly swappable with real chemist input without requiring model retraining.
• Multi-modal fusion.A multi-modal architecture integrates chemist knowledge and structural features through a gated multi-head cross-attention mechanism, enabling effective alignment between LLM-encoded expert insights and GNN-derived molecular representations.
• Empirical performance and interpretability.Empirical evaluations on MoleculeNet benchmarks demonstrate MolProphecy”s superior performance over prior methods, accompanied by interpretable predictions facilitated by model visualization techniques.
• Human validation.A proof-of-concept study shows that targeted review-and-correct interventions from medicinal chemists measurably enhance predictive accuracy, underscoring the feasibility of transitioning from proxy-HITL to a full HITL framework.

## Graphical abstract

**Figure:**
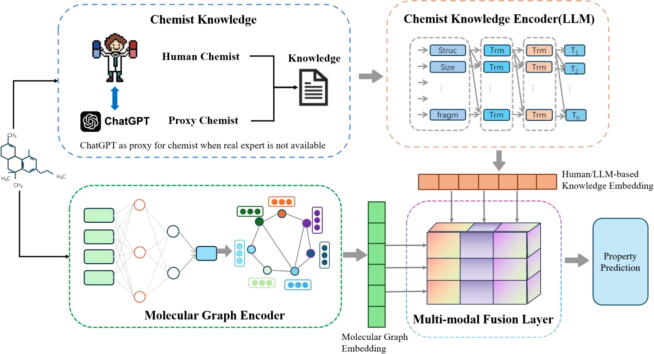

## Introduction

Drug discovery is a complex and resource-intensive process requiring deep expertise across chemistry, biology, and pharmacology. Medicinal chemists play a central role in designing molecules that maximize efficacy while minimizing side effects. Despite these efforts, the pipeline remains slow and heavily reliant on trial-and-error approaches, largely due to the difficulty of accurately predicting molecular properties in complex biological systems. The process often takes 10–15 years and costs between 1 and 2 billion dollars per approved drug [1]. Moreover, development costs have continued to rise despite technological advances, a trend commonly referred to as Eroom’s law [2]. A central bottleneck in this pipeline lies in the reliable prediction of molecular properties, which directly determines whether candidate compounds can progress successfully through subsequent stages of development [3].

Consequently, improving the accuracy and scalability of molecular property prediction has become a critical goal in modern drug discovery. In recent years, artificial intelligence (AI) has shown considerable promise in accelerating this process, particularly through large language models (LLMs). Foundational architectures such as BERT [4], GPT-3 [5], and LLaMA [6], together with domain-specific molecular pre-trained models, have enabled scalable molecular property prediction. However, most existing models focus solely on structural information (e.g. SMILES) and overlook the tacit, experience-based reasoning of medicinal chemists. This omission limits both predictive accuracy and interpretability, creating a critical gap that hinders the deployment of AI in real-world drug discovery.

To address this gap, we present MolProphecy, a proxy-human-in-the-loop (proxy-HITL) multi-modal framework that integrates chemist-inspired knowledge with graph-based molecular representations. By dynamically fusing these two complementary modalities through a gated multi-head cross-attention mechanism, MolProphecy achieves more accurate, robust, and interpretable predictions, aligning data-driven modeling with domain expertise.

This study has three primary objectives: (1) to design a scalable proxy-HITL framework that effectively incorporates chemist knowledge into molecular property prediction; (2) to investigate how multi-modal fusion enhances predictive accuracy and interpretability; and (3) to validate the generalizability of the framework across diverse benchmark datasets.

The contributions of this work are threefold. First, we propose a proxy-HITL design in which LLM-generated chemist rationales serve as a drop-in proxy for human expertise; the same knowledge interface can be replaced by real expert input without retraining. Second, we develop a multi-modal fusion architecture that aligns chemist knowledge with molecular graph features through gated cross-attention. Third, we conduct extensive experiments on nine MoleculeNet benchmarks, showing consistent performance improvements over prior methods, together with interpretability analyses that highlight the benefits of multi-modal fusion.

## Related work

### Molecular representation models

Pre-trained molecular representation models, which learn transferable chemical features from large-scale unlabeled data, have become central to computational chemistry by mitigating data scarcity and enhancing generalization. These models primarily follow two paradigms based on the input data type, namely, sequence-based and structure-based. Sequence-based models, such as ChemBERTa [7] and Mol-BERT [8], adapt Transformer architectures from natural language processing to SMILES strings, capturing chemical syntax and semantics. In parallel, structure-based approaches leverage Graph Neural Networks (GNNs), such as the foundational Graph Isomorphism Network (GIN) [9], to learn embeddings directly from the molecular graph and capture richer topological information. Among them, edge-aware extensions of GIN proposed by Hu et al. [10] have proven particularly effective for capturing fine-grained chemical interactions, motivating our adoption of a GIN-based architecture augmented with edge features.

### Molecular property prediction models

Improving molecular property prediction, a fundamental task in drug discovery, has motivated work on a range of data modalities. Early studies compared different molecular representations and their predictive value [11]. Methods such as Neural Message Passing [12] and Graph Attention Networks (GAT) [13] advanced graph-based learning, and large-scale pre-training approaches like GROVER [14] and Graphormer [15] used Transformer architectures to learn powerful structure-aware embeddings.

Recent research has turned to multi-modal frameworks. MolXPT [16] wraps molecules with textual context during generative pre-training, whereas SYN-FUSION [17] combines structural graphs with chemistry-guided information. Yang [18] proposed a multi-task aquatic toxicity prediction model based on multi-level feature fusion, demonstrating the benefits of integrating diverse feature sets. MulAFNet [19] integrates complementary molecular views through adaptive fusion, and MolPROP [20] unifies language and molecular graphs via joint representation learning.

Although these fusion models improve accuracy, they remain predominantly data-driven and provide limited chemical interpretability. This limitation highlights the need for frameworks that integrate qualitative domain expertise [21].

### LLMs and HITL in drug discovery

LLMs have recently emerged as powerful tools in chemistry, with platforms like ChemCrow [22] and Coscientist [23] demonstrating their capability to assist in complex tasks like synthesis planning. A comprehensive review by Chakraborty [24] further highlights the transformative potential of AI-enabled language models, including LLMs and multi-modal large language models (MLLMs), across various stages of drug discovery and development. Notably, studies show that models like GPT-4 can match or exceed the competencies of mid-level professional chemists on standardized exams and advanced reasoning benchmarks [25], [26], suggesting their potential to significantly augment chemical research [27], [28].

However, most systems use LLMs as external agents rather than structurally integrating their knowledge into predictive models. While HITL frameworks have been employed to align model behavior with expert preferences [29], [30], they rarely formalize domain knowledge within the model architecture itself. Recent work further shows that although LLMs are insufficient as standalone validators, combining them with automated methods and selective human oversight can achieve human-level quality with reduced manual effort [31].

## Materials and methods

### Overall architecture of MolProphecy

**Fig. 1:**
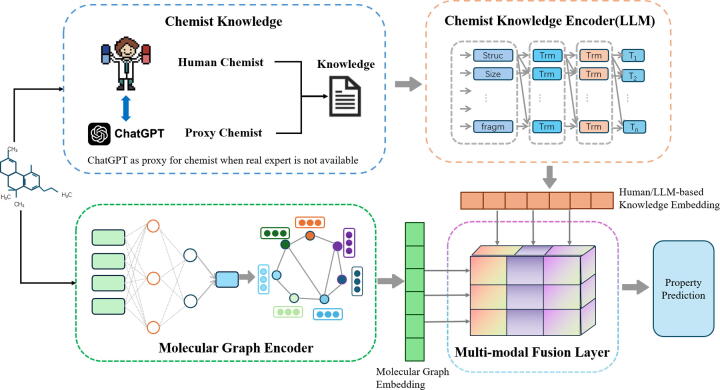
Overview of the proposed proxy-HITL framework for molecular property prediction. Chemist knowledge (collected from experts or generated by ChatGPT as a virtual proxy) and molecular structure features are encoded and fused via a multi-modal layer for downstream tasks. Trm refers to Transformer blocks within the LLaMA3-based Knowledge Encoder.

Motivated by the need to incorporate chemist reasoning, MolProphecy integrates domain knowledge and structural representations via a multi-modal design. As illustrated in Fig. 1, it comprises four components: (1) a chemist-knowledge generation module that collects knowledge insights from human chemist or ChatGPT; (2) a chemist-knowledge pathway that encodes these insights with an LLM; (3) a molecular-structure pathway that extracts graph-based features from molecular inputs; and (4) a multi-modal fusion module that integrates the two modalities through cross-attention.

The model is trained end-to-end for downstream property prediction tasks. We next describe how chemist-like knowledge is simulated and embedded, forming one of the two core modalities in our model.

### Chemist knowledge simulation via ChatGPT

Recent benchmarks such as GPQA [32] provide compelling evidence that LLMs are approaching or even surpassing human expert performance in structured scientific reasoning. While human experts average around 65% accuracy on graduate-level, Google-proof multiple-choice questions spanning biology, physics, and chemistry, LLMs like Grok-4 and o3-pro achieve 87.7% and 84.5%, respectively [33]. These results demonstrate that LLMs can serve as credible surrogates for expert-level reasoning in complex scientific domains. Nonetheless, the possibility of hallucinations or overly general insights remains, particularly in open-ended tasks. Importantly, such imperfections are not unique to LLMs; human experts also make errors and often rely on heuristics or incomplete information. What distinguishes our framework is its robustness to these imperfections. By explicitly integrating LLM-generated chemist-style insights as a distinct modality alongside structured molecular features, MolProphecy-Full exhibits the ability to mitigate and absorb hallucinated or vague reasoning.

**Fig. 2:**
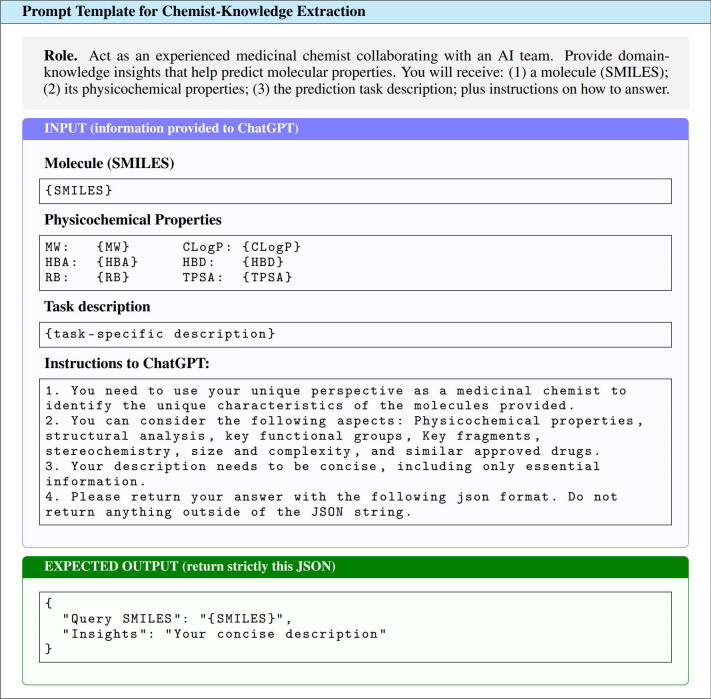
Overall layout of the prompt template used for chemist-knowledge extraction. Full task-specific prediction objectives are provided in Appendix A.

To generate scalable and consistent expert-level insights, we employ ChatGPT as a virtual proxy for medicinal chemists. A carefully designed structured prompt is used to elicit domain-specific reasoning grounded in molecular context. The framework is designed to be knowledge source-agnostic: real chemists can be seamlessly integrated into the same pipeline by providing insights in the same prompt format, enabling future HITL workflows without requiring retraining. The full prompt template is shown in Fig. 2. To ensure reproducibility, reference insights were generated under a deterministic decoding setting (temperature  = 0). Detailed generation and evaluation protocols are provided in Section 3.7.

**Fig. 3:**
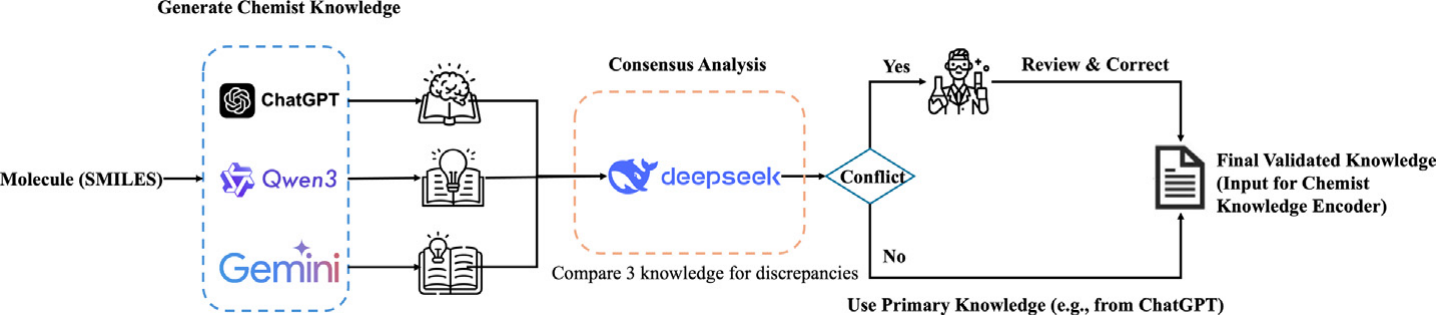
The human validation workflow. This process combines a multi-LLM consensus mechanism to identify high-uncertainty challenge cases with targeted review by human chemists to generate refined, high-quality knowledge for downstream tasks.

*Knowledge Source and Proxy-HITL.* In the present implementation, MolProphecy adopts a proxy-HITL design where chemist expertise is simulated by ChatGPT. To empirically validate this design and the framework’s ability to integrate real chemist expertise, we designed and executed a human validation workflow, as illustrated in Fig. 3. This workflow comprised two main stages.

First, we conducted a multi-LLM consensus analysis to identify challenge cases with high predictive uncertainty. For the BACE dataset, three distinct LLMs (ChatGPT, Qwen3 [34], and Gemini [35]) were prompted with the identical template shown in Fig. 2 to independently generate chemist-style insights. A fourth LLM (DeepSeek-Chat [36]) was then employed as a neutral judge to compare these outputs. The judge was instructed via a structured prompt to assess and flag molecules with significant discrepancies in key areas of chemical reasoning, including functional group identification, physicochemical property analysis, and ADMET heuristics. The full prompt for the judge LLM is provided in Appendix D.

In the second stage, a subset of these flagged challenge cases was submitted to experienced medicinal chemist for refinement under a review-and-correct paradigm, where they edited the conflicting LLM-generated knowledge. The finalized, human-corrected knowledge were then used for downstream evaluation without any model retraining.

### Chemist knowledge encoder

Following the generation of chemist-style reasoning via ChatGPT, we employ an LLM to encode the resulting chemist knowledge into rich, contextualized embeddings suitable for fusion with structural information. This module is specifically designed to capture high-level chemical reasoning, including structural interpretation, functional group recognition, physicochemical property estimation, and ADMET risk assessment. This approach avoids treating the input as generic text and instead preserves the domain-specific nature of the simulated expertise, ensuring the encoded knowledge aligns with our HITL objective.

The final chemist knowledge embedding $h_{\mathrm{chem}}\in R^{n\times d}$ is obtained by passing the tokenized chemist insight *X* through the LLaMA3 model. We use the resulting hidden states as contextualized token-level representations without any additional pooling, allowing subsequent fusion layers to learn optimal integration strategies.

(1) $$h_{\mathrm{chem}}=\mathrm{LLaMA}3(X)$$

### Molecular graph encoder

We adopt a GIN-based architecture augmented with edge features as the Molecular Graph Encoder to extract structural representations from a molecular graph G=(V,E), where *V* denotes the set of atoms and *E* represents the set of chemical bonds. Each atom v∈V is initially associated with a feature vector $h_{v}^{\left(0\right)}$, derived from categorical embeddings of atom type and chirality. Each bond e∈E is represented by its bond type and bond direction, which are embedded and combined during message passing. A detailed description of atom and bond features is provided in the Supplementary Information (Appendix C).

To capture bond-level interactions, we incorporate edge-conditioned message passing. At the *k*-th iteration (k=1,…,K), the node embedding $h_{v}^{\left(k\right)}$ is computed as:

(2) $$h_{v}^{\left(k\right)}=\mathrm{MLP}\left(h_{v}^{\left(k-1\right)}+\sum_{u\in N(v)}\left(h_{u}^{\left(k-1\right)}+e_{\mathrm{uv}}\right)\right)$$

where $e_{\mathrm{uv}}$ denotes the edge embedding between nodes *u* and *v*, constructed by summing learned embeddings for bond type and bond direction. Self-loops are explicitly added as additional edges with predefined edge types.

After *K* iterations, a graph-level molecular representation $h_{\mathrm{mol}}$ is obtained by applying a readout function to the final node embeddings:

(3) $$h_{\mathrm{mol}}=\mathrm{READOUT}(\{h_{v}^{\left(K\right)}|v\in V\})$$

In our implementation, the READOUT function is instantiated as mean pooling:

(4) $$h_{\mathrm{mol}}=\frac{1}{\left|V\right|}\underset{v\in V}{\sum }h_{v}^{\left(K\right)}$$

### Multi-modal fusion layer

**Fig. 4:**
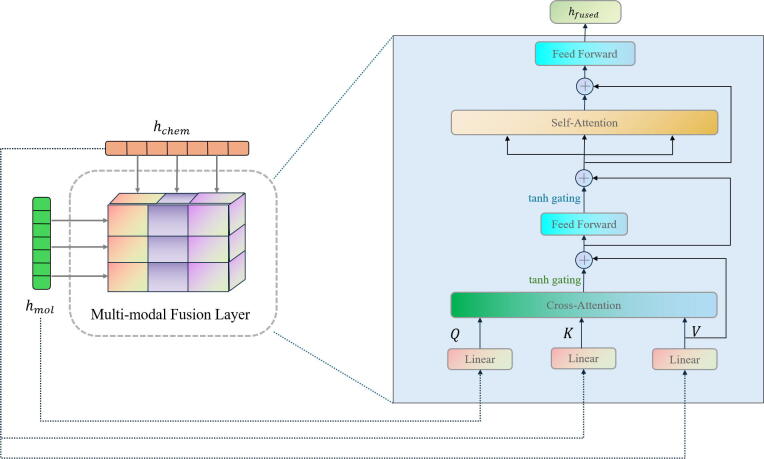
Architecture of the Multi-modal Fusion Layer. It illustrates the flow from molecular and chemist knowledge embeddings through multi-head cross-attention, gated feedforward, self-attention, and feedforward layers to produce the final fused representation.

The multi-modal fusion layer, illustrated in Fig. 4, employs a gated multi-head cross-attention mechanism. In this setup, the molecular graph embedding $h_{\mathrm{mol}}$ acts as the query, while the chemist knowledge embedding $h_{\mathrm{chem}}$ serves as both key and value. This asymmetric configuration reflects our design intuition: the molecular structure encodes the compound’s core identity, while chemist knowledge provides contextual guidance. By treating the structure as the querying entity, the model selectively attends to relevant expert insights, enriching the representation without overriding its intrinsic topological features. This mirrors how medicinal chemists typically analyze molecules, starting from structure and applying domain knowledge to infer potential behavior. Learnable gating functions further regulate the information flow, enabling controlled integration of chemist insights. This design is inspired by recent advances in cross-modal attention for molecular tasks [37].

Let $h_{\mathrm{mol}}\in R^{n\times d}$ denote the molecular graph embedding and $h_{\mathrm{chem}}\in R^{m\times d}$ denote the chemist knowledge embedding, where *n* and *m* are the number of molecular and chemist knowledge tokens respectively, and *d* is the embedding dimension.

(5) $$A_{\mathrm{cross}}=\mathrm{MultiheadAttention}(h_{\mathrm{mol}},h_{\mathrm{chem}},h_{\mathrm{chem}},\mathrm{mask}=M_{\mathrm{chem}})$$

where $M_{\mathrm{chem}}$ is a padding mask for the chemist knowledge input. The attended representation is modulated via a learnable gating parameter $\alpha_{\mathrm{xattn}}$, yielding an updated molecular representation:

(6) $$h_{\mathrm{mol}}\leftarrow h_{\mathrm{mol}}+\tanh(\alpha_{\mathrm{xattn}})\cdot A_{\mathrm{cross}}$$

Following the cross-attention step, a gated feedforward layer is applied:

(7) $$h_{\mathrm{mol}}\leftarrow h_{\mathrm{mol}}+\tanh(\alpha_{\mathrm{dense}})\cdot \mathrm{FFN}(h_{\mathrm{mol}})$$

where FFN denotes a feedforward neural network with ReLU activation. To enhance the contextual understanding within the molecular representation, a self-attention module is then applied:

(8) $$A_{\mathrm{self}}=\mathrm{SelfAttn}(h_{\mathrm{mol}},h_{\mathrm{mol}},h_{\mathrm{mol}},\mathrm{mask}=M_{\mathrm{mol}})$$

resulting in a further refined embedding:

(9) $$h_{\mathrm{mol}}\leftarrow h_{\mathrm{mol}}+A_{\mathrm{self}}$$

Lastly, an additional feedforward layer produces the final fused representation:

(10) $$h_{\mathrm{fused}}=h_{\mathrm{mol}}+\mathrm{FFN}(h_{\mathrm{mol}})$$

which integrates both structural and chemist knowledge features.

### Prediction and training strategy

The fused representation $h_{\mathrm{fused}}$ is fed into a multi-layer perceptron (MLP) for downstream molecular property prediction. The configuration of the output layer and the choice of loss function are determined by the specific task type.

For classification tasks, the output is a probability score computed using a sigmoid function:

(11) y^=σ(Whfused+b)

with binary cross-entropy (BCE) loss:

(12) Lpred=-1N∑i=1Nyilogy^i+(1-yi)log(1-y^i)

For regression tasks, a linear projection produces continuous outputs:

(13) y^=Whfused+b

with mean squared error (MSE) loss:

(14) Lpred=1N∑i=1N(yi-y^i)2

The model is trained end-to-end using only the prediction loss $L_{\mathrm{pred}}$. During training, the LLaMA3-based Chemist Knowledge Encoder remains fixed, while the Molecular Graph Encoder, the fusion layer, and the prediction head are jointly optimized. Freezing the LLM encoder reduces computational cost and mitigates overfitting risks on limited datasets, while still allowing effective transfer of domain knowledge via its pre-trained representations. This design choice is also consistent with prior work demonstrating that frozen LLMs can serve as strong semantic feature extractors for downstream tasks[38].

### Experimental setup

All experiments were implemented in PyTorch and executed on an NVIDIA RTX 4090 GPU. Model training was performed using the Adam optimizer with an initial learning rate of $3\times 10^{-4}$, combined with a learning rate scheduler that reduces the rate by a factor of 0.5 upon plateau (patience of 5 epochs). A maximum of 100 epochs was allowed with early stopping based on validation performance. The training batch size was set to 128, with dropout 0.5 applied to the final MLP layer and a weight decay coefficient of $10^{-3}$. To ensure statistical reliability, all experiments were repeated five times with random seeds {0,1,2,3,4}, and the final results are reported as the mean ± standard deviation across these runs.

For the generation of chemist-style insights, we used ChatGPT under a deterministic decoding configuration (temperature = 0) during training-time reference construction, ensuring reproducibility across runs. For consistency analysis, we deliberately adopted a stochastic decoding configuration (temperature = 0.7) without fixing seeds, to capture natural variability in LLM outputs. Semantic similarity scoring was performed using a separate LLM (DeepSeek-Chat, temperature = 0), ensuring stable and reproducible evaluation. The exact prompts used for similarity scoring are provided in the Supplementary Information (Appendix B).

### Evaluation metrics

Model performance was quantified using metrics tailored to each task type. For classification tasks, we employ the Area Under the Receiver Operating Characteristic Curve (AUROC), which measures the model’s ability to discriminate between positive and negative classes:

(15) $$\mathrm{AUROC}=\int_{0}^{1}\mathrm{TPR}(\mathrm{FPR})\;d(\mathrm{FPR})$$

where $\mathrm{TPR}=\frac{\mathrm{TP}}{\mathrm{TP}+\mathrm{FN}}$ is the True Positive Rate and $\mathrm{FPR}=\frac{\mathrm{FP}}{\mathrm{FP}+\mathrm{TN}}$ is the False Positive Rate.

In addition, we report the Area Under the Precision–Recall Curve (AUC-PR), which is particularly informative under class-imbalanced conditions such as BBBP, HIV, SIDER, ClinTox, and Tox21:

(16) $$\mathrm{AUC}-\mathrm{PR}=\int_{0}^{1}\mathrm{Precision}(\mathrm{Recall})\;d(\mathrm{Recall})$$

where $\mathrm{Precision}=\frac{\mathrm{TP}}{\mathrm{TP}+\mathrm{FP}}$ and $\mathrm{Recall}=\frac{\mathrm{TP}}{\mathrm{TP}+\mathrm{FN}}$ (equivalent to TPR defined above).

For regression tasks, our performance metric is the Root Mean Squared Error (RMSE), where lower RMSE indicates better predictive accuracy:

(17) RMSE=1n∑i=1n(yi-y^i)2

where $y_{i}$ represents the true value, y^i represents the predicted value, and *n* is the number of samples.

This unified benchmarking setup enables a comprehensive and fair comparison across methods and tasks, supporting the robustness and generalizability of our proposed fusion framework.

## Results

### Datasets

The proposed model, MolProphecy, was evaluated on nine benchmark datasets from MoleculeNet [39], selected to cover both regression and classification tasks of varying size and difficulty. These include three regression datasets (FreeSolv, ESOL, Lipophilicity) and six classification datasets (BBBP, BACE, HIV, SIDER, ClinTox, Tox21). Key statistics for these datasets are summarized in Table 1.

**Table 1:** Descriptions of the datasets used in this study.

**Category**	**Dataset**	**# Molecules**	**# Tasks**	**Task Type**
Physical	BBBP	2039	1	Classification
	SIDER	1427	27	Classification
	ClinTox	1478	2	Classification
	Tox21	7831	12	Classification
Physical Chemistry	FreeSolv	642	1	Regression
	ESOL	1128	1	Regression
	Lipophilicity	4200	1	Regression
Biophysics	BACE	1513	1	Classification
	HIV	41127	1	Classification

**Fig. E.10:**
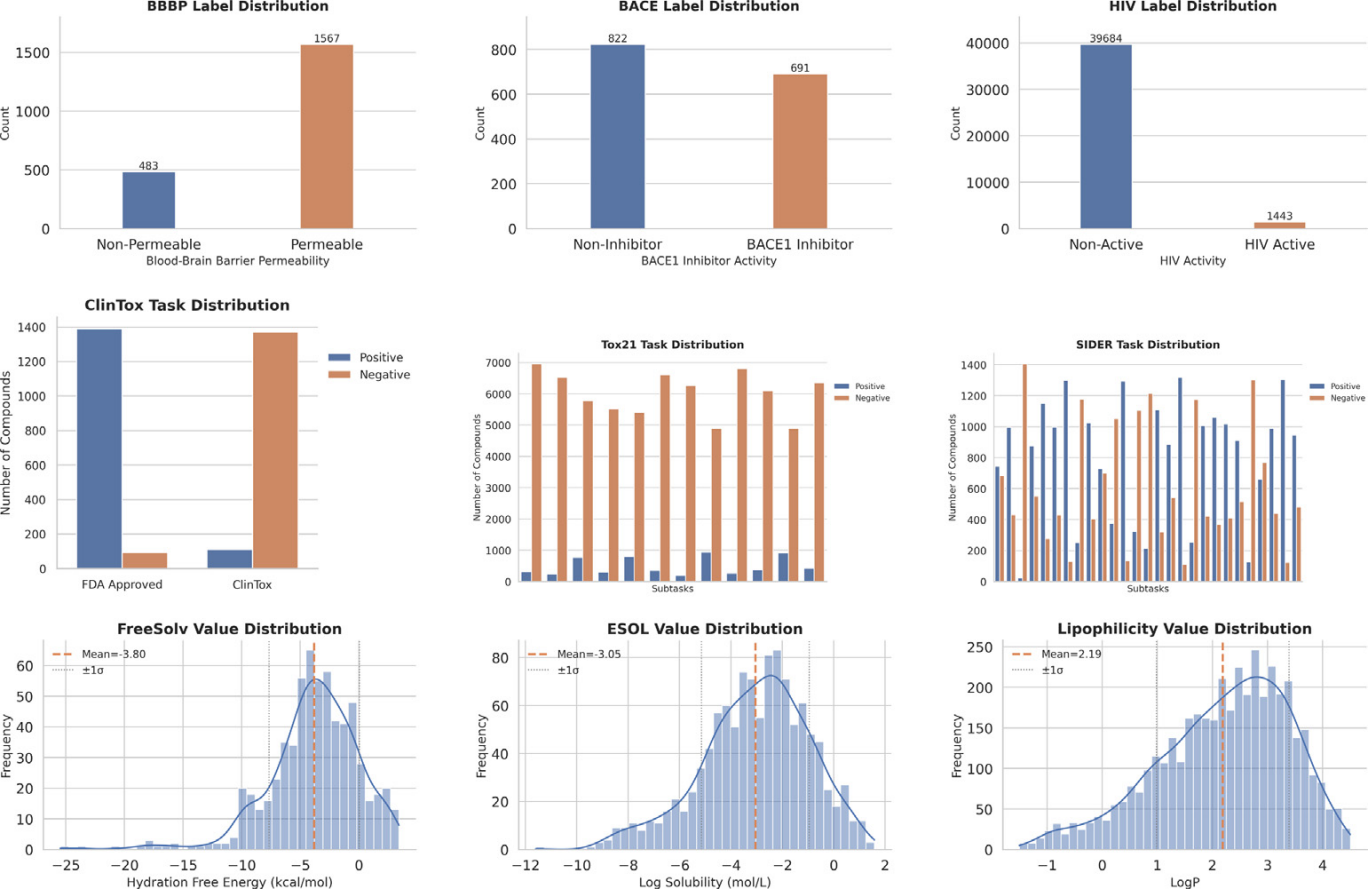
Distribution characteristics of the nine MoleculeNet datasets used in this study. Top row: BBBP, BACE, HIV. Middle row: SIDER, ClinTox, Tox21. Bottom row: FreeSolv, ESOL, Lipophilicity. Classification datasets highlight varying degrees of label imbalance, while regression datasets exhibit wide ranges with outliers.

For regression datasets, we followed the MoleculeNet protocol and applied random splits with an 8:1:1 ratio for training, validation, and test sets. For classification datasets, we adopted scaffold splits [40], also with an 8:1:1 ratio, to prevent structural information leakage across sets. Dataset distribution characteristics highlight several inherent challenges, including severe class imbalance in SIDER, ClinTox, and Tox21, as well as the wide, outlier-rich property distributions in FreeSolv, ESOL, and Lipophilicity. These characteristics underscore the need for models that exhibit robustness to both label imbalance and numerical instability. Full distribution visualizations and detailed splitting strategies are provided in Appendix E (Table E.12 and Fig. E.10).

**Fig. E.11:**
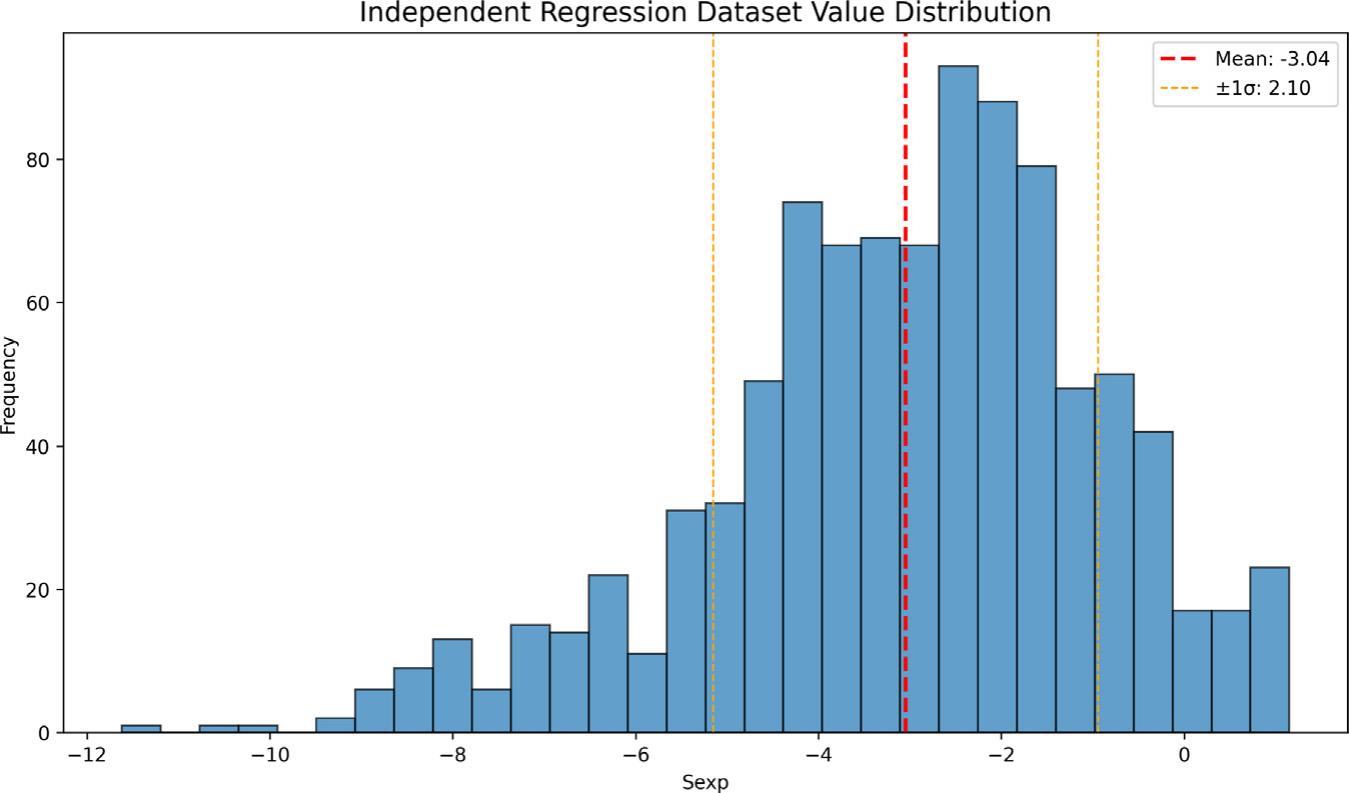
Value distribution of the independent solubility dataset after preprocessing. This distribution highlights the chemical diversity of the 949 curated molecules retained for external validation (Section 4.3).

*External Validation Dataset.* An independent solubility dataset curated by Ulrich [41] was used to evaluate MolProphecy beyond the MoleculeNet benchmarks and to mitigate concerns of potential data exposure during LLM pretraining. After removing overlaps with all MoleculeNet datasets, 949 unique molecules were retained from the original 1291 curated entries. The dataset was originally proposed as an external benchmark for water solubility prediction and provides a realistic testbed for assessing the generalization capability of MolProphecy. Details of preprocessing and overlap removal are provided in the Supplementary Information (Table E.13, Fig. E.11).

### Performance comparison with baseline models

MolProphecy was compared against a broad set of baselines, including GNNs (e.g., D-MPNN, GAT), SMILES-based language models (ChemBERTa), and various advanced architectures, including large-scale pre-trained models like GROVER and Graphormer, and recent multi-modal approaches such as SYN-FUSION and MolPROP.

For regression tasks (Table 2), MolProphecy achieved the best performance on FreeSolv with an RMSE of 0.796±0.09, representing a 9.1% reduction relative to SYN-FUSION (0.876±0.04). On Lipophilicity, MolHGT achieved the strongest performance (0.536±0.032), while MolProphecy remained competitive at 0.657±0.07. Across all regression datasets, MolProphecy consistently demonstrated stable accuracy.

**Table 2:** Performance comparison on regression tasks (RMSE ↓). Bold indicates the best performance.

**Method**	FreeSolv	ESOL	Lipophilicity
GAT [11]	3.14	1.41	0.89
D-MPNN [11]	2.18 ± 0.91	0.98 ± 0.26	0.75 ± 0.05
ChemBERTa [7]	2.05	0.889	0.798
GROVER [14]	1.99 ± 0.07	1.10 ± 0.18	0.82 ± 0.01
Graphormer [15]	2.09 ± 0.75	0.93 ± 0.04	1.10 ± 0.39
MolHGT [42]	0.929 ± 0.121	**0.518** ± **0.021**	**0.536** ± **0.032**
MolCLR [43]	2.20 ± 0.20	1.11 ± 0.01	0.60 ± 0.08
SYN-FUSION [17]	0.876 ± 0.04	0.89 ± 0.02	0.72 ± 0.01
MolPROP [20]	1.70 ± 0.09	0.777 ± 0.02	0.733 ± 0.02
**MolProphecy (Ours)**	**0.796** ± **0.09**	0.693 ± 0.006	0.657 ± 0.07

For classification tasks (Table 3), MolProphecy ranked among the top-performing methods. On BACE, it achieved an AUROC of 0.938±0.003, representing a 5.4% gain over the best baseline. On ClinTox, it reached 0.957±0.004, outperforming previous models. On SIDER, MolProphecy obtained 0.709±0.009, exceeding SYN-FUSION (0.699±0.013). For HIV, MolCLR reported a slightly higher AUROC of 0.806±0.011 compared with MolProphecy’s 0.774±0.015, though the two results were close within the margin of variability.

**Table 3:** Performance comparison on classification tasks (AUROC ↑). Bold indicates the best performance. Since datasets such as BBBP, HIV, SIDER, ClinTox, and Tox21 are highly imbalanced, we further report AUC-PR results in Table 4

**Method**	BACE	BBBP	HIV	SIDER	ClinTox	Tox21
GAT [11]	0.579	0.580	–	–	0.541	–
D-MPNN [11]	0.853 ± 0.053	0.712 ± 0.038	0.750 ± 0.021	0.632 ± 0.023	0.905 ± 0.053	0.689 ± 0.013
ChemBERTa [7]	0.799	0.728	–	–	0.872	–
GROVER [14]	–	–	0.682 ± 0.011	0.658 ± 0.023	0.944 ± 0.021	–
Graphormer [15]	–	–	0.789 ± 0.009	0.620 ± 0.012	0.881 ± 0.038	–
MolHGT [42]	0.857 ± 0.008	0.738 ± 0.003	0.780 ± 0.026	0.676 ± 0.013	0.747 ± 0.021	–
MolCLR [43]	0.890 ± 0.003	0.736 ± 0.005	**0.806** ± **0.011**	0.680 ± 0.011	0.932 ± 0.017	0.798 ± 0.007
SYN-FUSION [17]	0.805 ± 0.011	0.742 ± 0.009	0.763 ± 0.013	0.699 ± 0.013	0.947 ± 0.002	0.751 ± 0.006
MolPROP [20]	0.687 ± 0.020	0.631 ± 0.023	–	–	0.933 ± 0.035	–
**MolProphecy (Ours)**	**0.938** ± **0.003**	**0.899** ± **0.007**	0.774 ± 0.015	**0.709** ± **0.009**	**0.957** ± **0.004**	**0.818** ± **0.007**

In addition to the reported values from prior studies, we also re-implemented several widely used open-source baselines (D-MPNN, ChemBERTa, and MolPROP) under identical random seeds, data splits, and training protocols to ensure a fair comparison. For regression tasks, MolProphecy achieved clear improvements over re-implemented baselines across all datasets, reducing RMSE to 0.796±0.09 on FreeSolv compared with 1.63±0.11 for MolPROP and 1.89±0.41 for D-MPNN (see Table F.14 in the Appendix F). For classification tasks, the corresponding re-implementation results are summarized in Table 4. Importantly, while prior benchmarks primarily reported AUROC, we additionally introduce AUC-PR as a complementary metric that is more informative under class imbalance. For example, under identical splits, MolProphecy achieved 0.385±0.024 AUC-PR on HIV compared with 0.193±0.019 for MolPROP, and 0.843±0.004 on BBBP compared with 0.697±0.020 for D-MPNN, confirming the robustness of our framework under skewed label distributions.

**Table 4:** Comparison of AUROC and AUC-PR on five MoleculeNet classification datasets. The results for baseline models are based on our re-implementation under identical protocols to ensure a fair comparison. For imbalanced datasets, AUC-PR provides a more informative evaluation.

(a) Performance on BBBP, HIV, and SIDER datasets.						
**Model**	**BBBP**		**HIV**		**SIDER**	
	AUROC	AUC-PR	AUROC	AUC-PR	AUROC	AUC-PR

D-MPNN	0.71 ± 0.031	0.697 ± 0.02	0.744 ± 0.017	0.152 ± 0.011	0.641 ± 0.018	0.596 ± 0.010
ChemBERTa	0.731 ± 0.009	0.620 ± 0.020	0.713 ± 0.013	0.119 ± 0.009	0.586 ± 0.024	0.552 ± 0.011
MolPROP	0.635 ± 0.017	0.648 ± 0.021	0.698 ± 0.015	0.193 ± 0.019	0.643 ± 0.022	0.575 ± 0.014
MolProphecy	**0.899**±**0.007**	**0.843**±**0.004**	**0.774**±**0.015**	**0.385**±**0.024**	**0.709**±**0.009**	**0.620**±**0.007**

(b) Performance on ClinTox and Tox21 datasets.				
**Model**	**ClinTox**		**Tox21**	
	AUROC	AUC-PR	AUROC	AUC-PR

D-MPNN	0.90 ± 0.044	0.953 ± 0.021	0.691 ± 0.018	0.429 ± 0.034
ChemBERTa	0.863 ± 0.071	0.820 ± 0.013	0.662 ± 0.010	0.207 ± 0.007
MolPROP	0.935 ± 0.03	0.919 ± 0.009	0.727 ± 0.018	0.294 ± 0.023
MolProphecy	**0.957**±**0.004**	**0.967**±**0.002**	**0.818**±**0.007**	**0.554**±**0.005**

### External validation on an independent dataset

To further assess MolProphecy’s generalization ability, we evaluated its performance on the independent solubility dataset. We compared our model against representative baselines (D-MPNN, ChemBERTa, and MolPROP) under identical training protocols. As shown in Table 5, MolProphecy achieved the lowest prediction error (RMSE ↓), demonstrating enhanced robustness and transferability to chemical domains not covered in MoleculeNet.

**Table 5:** Performance on the independent solubility dataset. Bold values indicate the best performance.

**Method**	**RMSE** ↓
D-MPNN [11]	0.858 ± 0.034
ChemBERTa [7]	0.764 ± 0.069
MolPROP [20]	0.797 ± 0.017
**MolProphecy (Ours)**	**0.651**±**0.046**

### Ablation studies

The contribution of each component in MolProphecy was quantified through a series of ablation experiments. These studies analyze the role of individual modalities, the source of chemist knowledge, and the choice of encoder, providing a comprehensive understanding of how different design decisions affect model performance.

*Modality Ablation.* We first evaluated the contribution of each modality (Table 6). Removing either the chemist knowledge encoder (MolProphecy-GIN) or the molecular graph encoder (MolProphecy-Chem) leads to a clear drop in performance, especially for BACE where the AUROC decreases from 0.938±0.003 to 0.597±0.008 when using only chemist knowledge. These results confirm that structural features and chemist insights are complementary, and their fusion unlocks the full predictive potential of the framework.

**Table 6:** Ablation study results on four benchmark datasets. Regression performance is reported as RMSE (↓) under a random split, and classification performance as AUROC (↑) under scaffold splits. Bold values indicate the best performance for each dataset.

**Dataset**	**MolProphecy-Chem**	**MolProphecy-GIN**	**MolProphecy-Full**
FreeSolv (RMSE ↓)	1.695 ± 0.13	0.968 ± 0.09	**0.796**±**0.09**
BACE (AUROC ↑)	0.597 ± 0.008	0.861 ± 0.005	**0.938**±**0.003**
SIDER (AUROC ↑)	0.501 ± 0.011	0.631 ± 0.008	**0.709**±**0.009**
ClinTox (AUROC ↑)	0.781 ± 0.01	0.799 ± 0.007	**0.957**±**0.004**

*Chemist Knowledge Source Ablation.* We next examined whether the advantage of ChatGPT-generated chemist knowledge merely reflects simple molecular descriptors or can also be achieved by other general LLMs. To test the first possibility, we replaced ChatGPT outputs with six RDKit-calculated properties (molecular weight, CLogP, hydrogen bond acceptors, hydrogen bond donors, rotatable bonds, and TPSA) [44]. As shown in Table 7, MolProphecy-RDKit yields an RMSE of 1.016±0.09 on FreeSolv, notably higher than the 0.796±0.09 achieved by MolProphecy-Chem. On BACE, MolProphecy-RDKit reaches an AUROC of 0.892±0.005, compared to 0.938±0.003 for MolProphecy-Chem. These results confirm that ChatGPT provides richer chemist-style reasoning that goes beyond conventional descriptors.

**Table 7:** Ablation study replacing ChatGPT-generated chemist knowledge with RDKit descriptors. Results are reported on FreeSolv (RMSE ↓) and BACE (AUROC ↑).

**Model Variant**	**FreeSolv (RMSE** ↓**)**	**BACE (AUROC** ↑**)**
MolProphecy-RDKit	1.016 ± 0.09	0.892 ± 0.005
MolProphecy-Chem (ChatGPT)	**0.796**±**0.09**	**0.938**±**0.003**

We further compared ChatGPT with Tx-Gemma [45], a representative chemistry-specific LLM trained on molecular corpora. Both models were prompted with the same template to generate chemist-style knowledge. As shown in Table 8, ChatGPT achieved stronger performance, reaching 0.796±0.09 RMSE on FreeSolv compared to 0.983±0.07 with Tx-Gemma, and 0.938±0.003 AUROC on BACE compared to 0.901±0.006. These results suggest that while domain-specific LLMs like Tx-Gemma may excel at recalling structural statistics, the broader reasoning capabilities of a general-purpose model like ChatGPT generate insights that are more informative for this downstream prediction task. This finding provides supporting evidence for our selection of ChatGPT as the default knowledge generator in our study.

**Table 8:** Performance comparison between ChatGPT and Tx-Gemma as chemist knowledge generators. Both models were prompted with the same template to generate chemist insights.

**Knowledge Generator**	**FreeSolv (RMSE** ↓**)**	**BACE (AUROC** ↑**)**
Tx-Gemma	0.983 ± 0.07	0.901 ± 0.006
ChatGPT	**0.796**±**0.09**	**0.938**±**0.003**

*Chemist Knowledge Encoder Ablation.* We also conducted an ablation study to evaluate whether the choice of encoder for chemist knowledge embedding affects performance. While ChatGPT generates chemist knowledge, the encoder transforms these insights into vector representations compatible with molecular graph features. To test whether a simpler encoder suffices, we replaced the LLaMA encoder with a BERT encoder and compared results on two representative datasets (FreeSolv for regression and BACE for classification). As shown in Table 9, LLaMA achieved 0.796±0.09 RMSE on FreeSolv and 0.938±0.003 AUROC on BACE, outperforming BERT (1.024±0.08 RMSE and 0.903±0.003 AUROC). These results indicate that a stronger encoder preserves richer semantic information and enables more effective fusion with molecular graphs.

**Table 9:** Comparison of different encoders for chemist knowledge embedding.

**Chemist Knowledge Encoder**	**FreeSolv (RMSE** ↓**)**	**BACE (AUROC** ↑**)**
BERT	1.024 ± 0.08	0.903 ± 0.003
LLaMA	**0.796**±**0.09**	**0.938**±**0.003**

### Validating the Proxy-HITL framework: a proof-of-concept study

We conducted a targeted validation study to empirically validate our proxy-HITL concept and to assess the impact of integrating real chemist expertise. Using the multi-LLM consensus workflow described in Section 3.2, we first identified a stress-test subset of BACE molecules where LLM-generated insights were conflicting, indicating high uncertainty. These challenge cases were then reviewed and corrected by experienced medicinal chemists.

The performance of MolProphecy on this challenging 62-molecule subset, both before and after human intervention, is reported in Table 10. The results show that replacing the conflicting LLM insights with the human-refined versions led to a consistent and measurable improvement in predictive performance. Specifically, the AUROC score increased from 0.871 to 0.889, and accuracy rose from 0.793 to 0.815. While the absolute performance on this intentionally difficult subset is, as expected, lower than on the full BACE test set (0.938 AUROC), the key finding is the measurable gain derived from targeted human expertise. Table 11 provides representative cases where this human review successfully resolved critical conflicts in chemical reasoning.

**Table 10:** Effect of human chemist refinement on MolProphecy’s performance for a subset of BACE challenge cases.

**Knowledge Source for Challenge Cases**	**AUROC (**↑**)**	**Accuracy (**↑**)**
Conflicting LLM-generated insights	0.871	0.793
Chemist-refined insights	**0.889**	**0.815**

**Table 11:** Representative challenge cases where human review resolves conflicting LLM insights on functional group identification and physicochemical reasoning.

**Molecule (SMILES)**	**LLM-conflicting insights**	**Human-refined insight**
S(=O)(=O)(N(C) c1cc(cc(c1) C(=O) NC(C(O) CC(C(=O) NC(C(C) C) C(=O) NCc1ccc(F) cc1) C) COCc1cc(F) cc(F) c1) C(=O) NC(C) c1ccccc1) C	**ChatGPT**: “moderate to low hydration”	Corrected: “polar sulfonamide dominates; hydration moderate; permeability limited”
	**Gemini**: “relatively unfavorable hydration”	
	**Qwen3**: “more negative hydration free energy”	

Fc1cc(cc(F) c1) CC(NC(=O) C) C(O) C[NH2+]C1(CCCN(C1) C(=O) C) c1cc(ccc1) C(C)(C) C	**ChatGPT**: “quaternary ammonium”	Corrected: “protonated tertiary amine; hydrophilic; may limit CNS penetration”
	**Gemini**: “protonated primary amine”	
	**Qwen3**: “guanidinium-like cation”	

While this proof-of-concept study was conducted on a limited scale, it yields two important findings. First, the multi-LLM consensus mechanism is effective at isolating challenging molecules where model uncertainty is high. Second, and more critically, it demonstrates that targeted intervention from human chemists can measurably enhance predictive accuracy without requiring any model retraining, providing evidence for the feasibility of the proxy-HITL workflow.

### Robustness and interpretability

We further assessed the robustness of MolProphecy to variability and potential hallucination in LLM-generated chemist knowledge. Training-time insights were generated with deterministic decoding, while stochastic decoding produced 10 variants per molecule. Semantic similarity between each variant and its reference was scored by a separate evaluator LLM (see Appendix B). As shown in Fig. 9, median similarity scores remained above 4.0 across datasets, confirming that MolProphecy is robust to prompt phrasing and sampling variability.

**Fig. 5:**
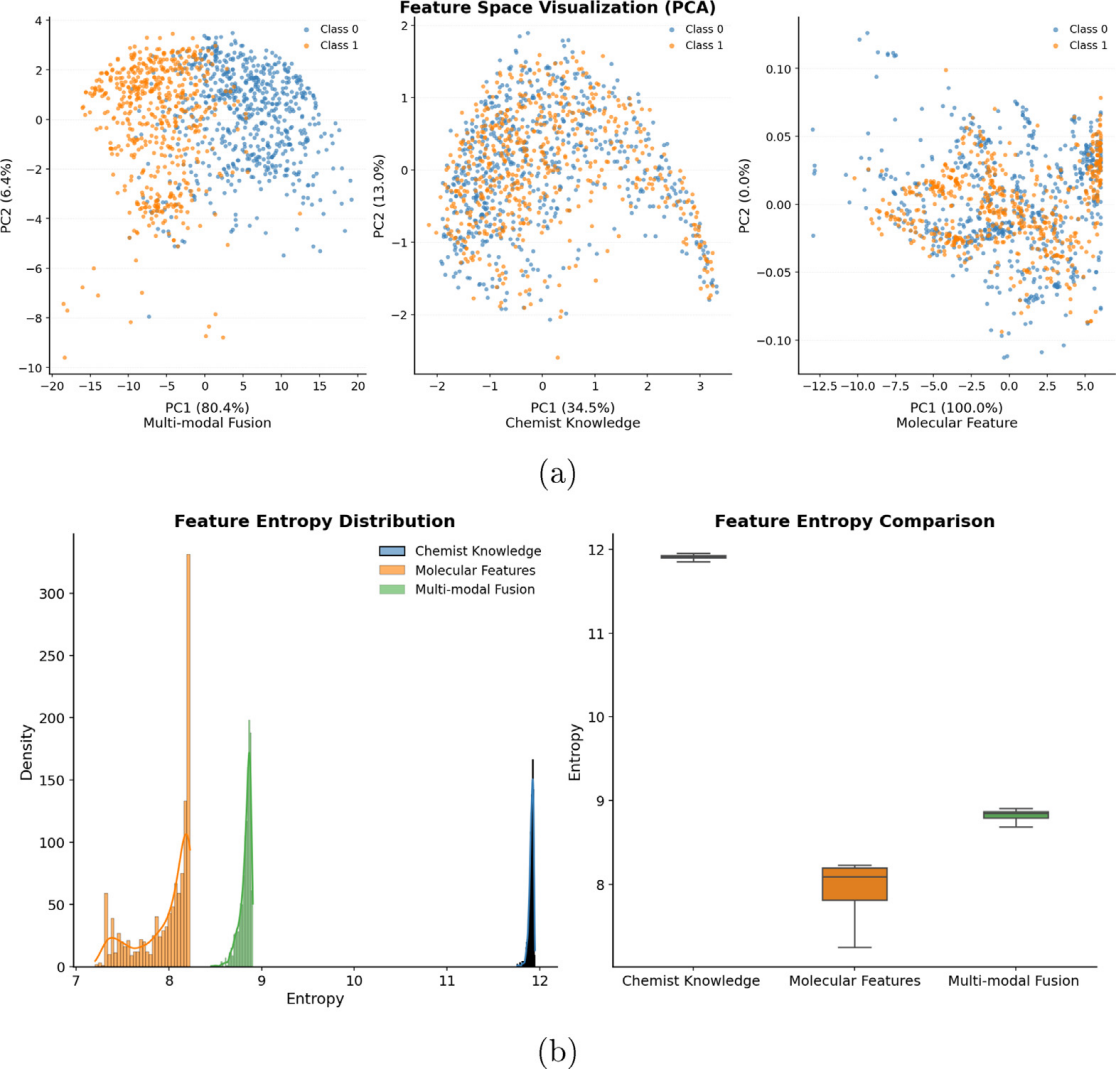
Feature analysis on the BACE dataset comparing MolProphecy-Full with unimodal variants. (a) PCA projections of learned representations, showing clearer class separation for MolProphecy-Full. (b) Entropy distributions, where fusion features exhibit intermediate entropy and low variance.

**Fig. 6:**
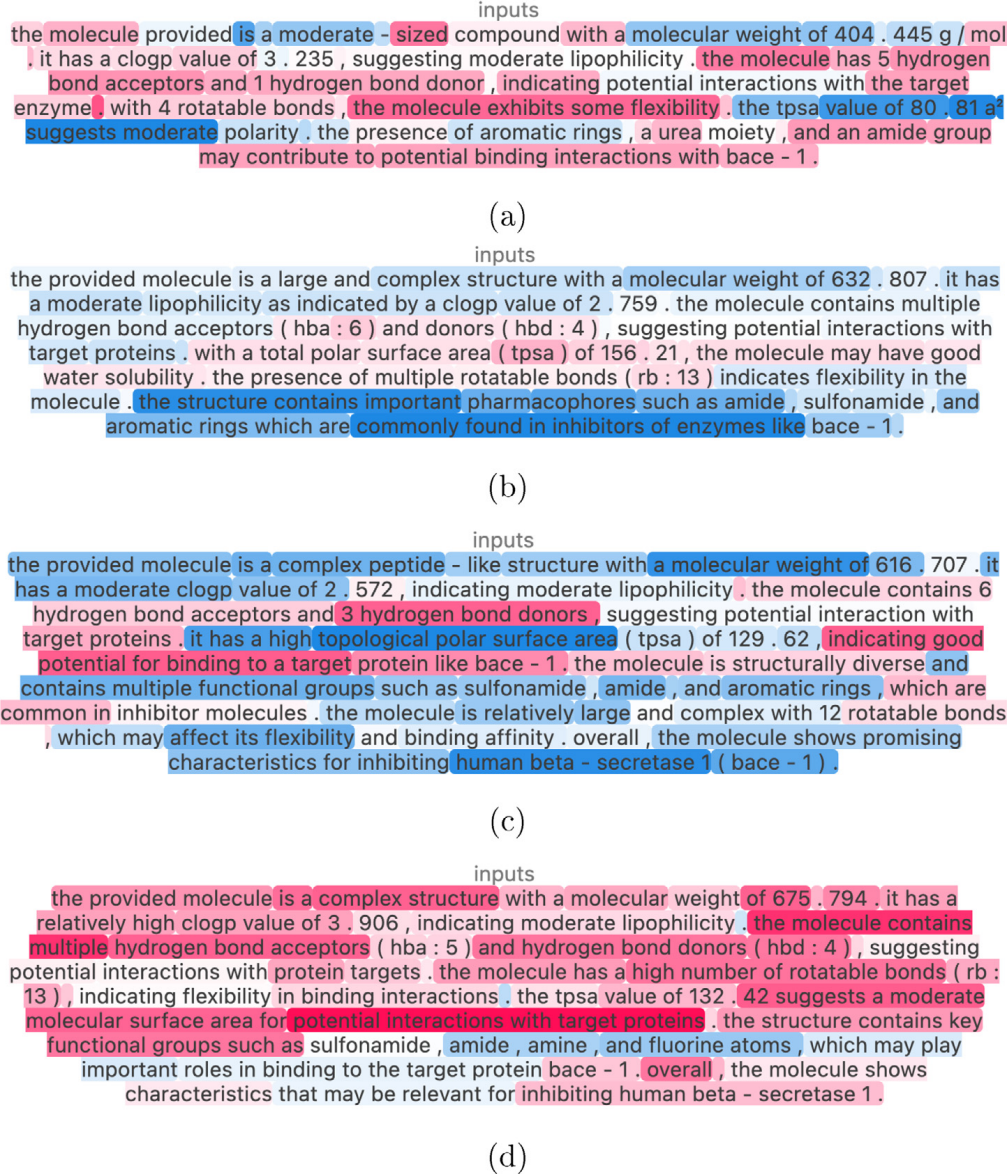
Token-level SHAP visualizations for MolProphecy on the BACE dataset. Highlighted tokens indicate contributions to the predicted class (blue  = positive, red  = negative). The results show that the model consistently attends to chemically meaningful descriptors.

**Fig. 7:**
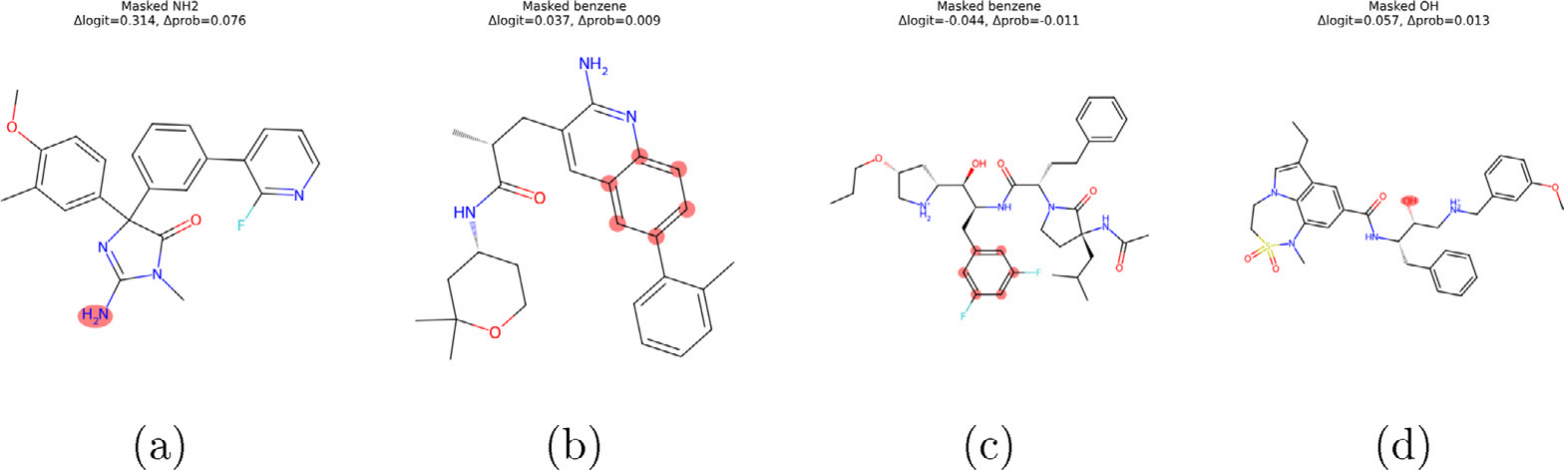
Case studies of SME on four representative BACE inhibitors. Each panel (a–d) corresponds to a single inhibitor, showing two structures for comparison: the baseline molecule on the right, and the result of masking a key functional group on the left (highlighted in red). The Δprob value quantifies the change in predicted probability of BACE inhibition after masking. Positive Δprob values indicate that these highlighted groups are correctly identified by the model as positive contributors to the predicted activity.

**Fig. 8:**
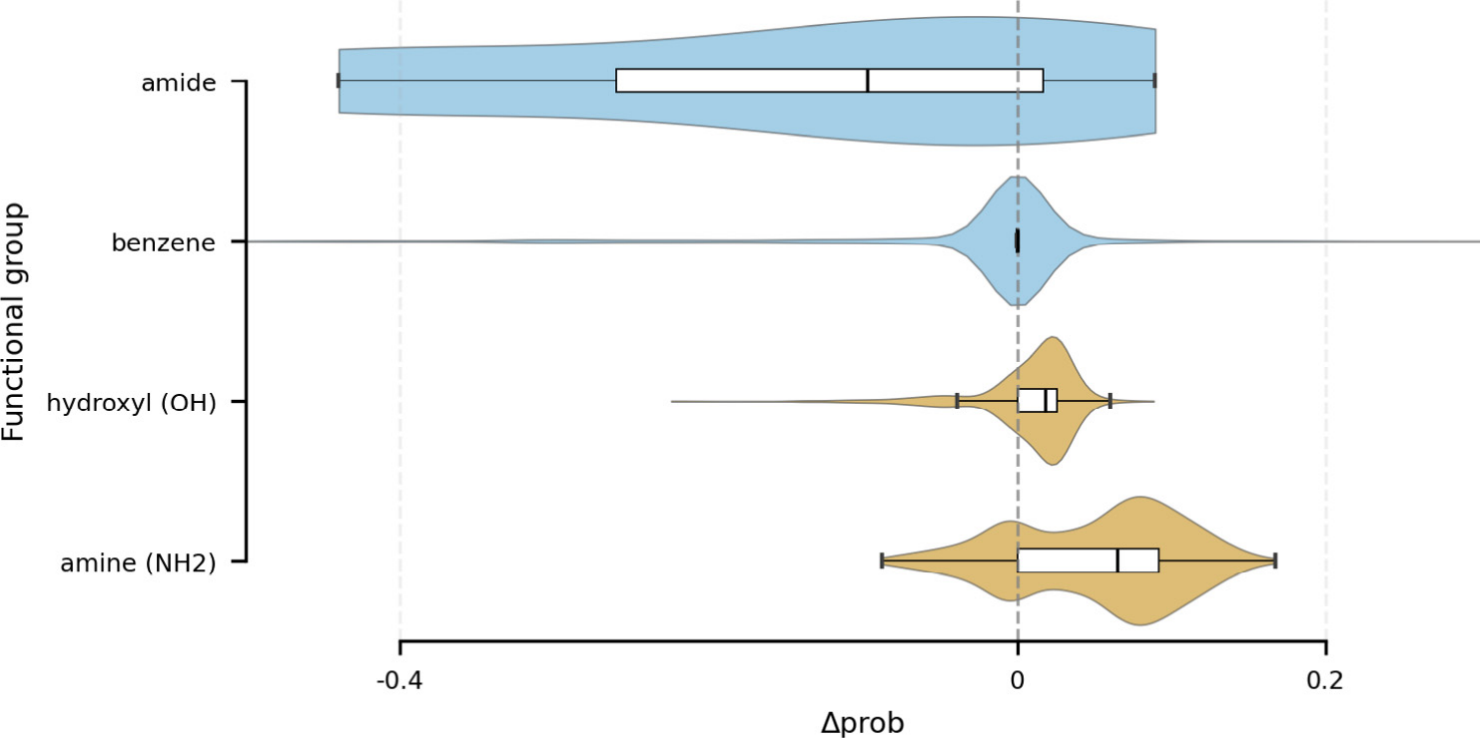
Aggregate analysis of functional group contributions on the BACE dataset using SME [47]. The violin plots display the distribution of Δprob values for key functional groups across the dataset. Δprob represents the change in predicted probability of BACE inhibition when a group is masked; a positive value indicates a favorable contribution to activity. Blue highlights groups that, on average, are favorable for activity (mean Δprob  > 0), while orange highlights groups that are, on average, unfavorable or neutral. The width of each violin reflects the density of molecules at that Δprob value, revealing that the influence of some groups (e.g., the broad distribution for ’amide’) is highly context-dependent.

To illustrate interpretability, we analyzed fused representations via PCA and entropy (Fig. 5), which revealed more discriminative and balanced feature spaces than unimodal counterparts. Attribution studies further highlight that the framework grounds its predictions in chemically meaningful features: SHAP [46] emphasized physicochemical descriptors (e.g., molecular weight, aromaticity) showed in Fig. 6, while Substructure Masking Explanation (SME) [47] pinpointed functional groups such as amides and hydroxyls (Fig. 7, Fig. 8). These analyses demonstrate that fusion not only enhances predictive accuracy but also aligns model reasoning with established chemical principles.

**Fig. 9:**
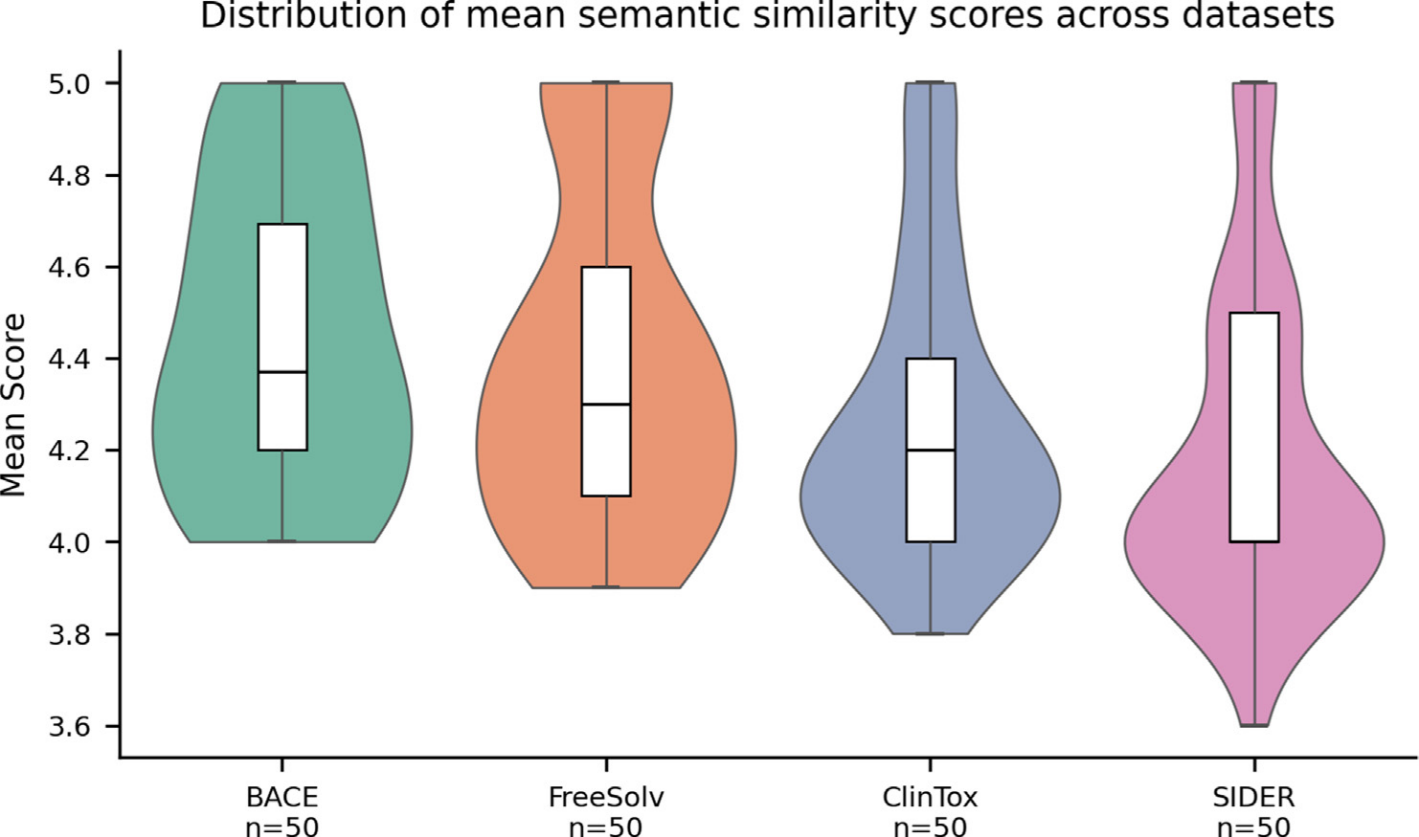
Distribution of per-molecule mean semantic similarity scores across four benchmark datasets. Each score reflects the average similarity between 10 stochastically generated insights and the deterministic reference for the same molecule. Detailed decoding and evaluation settings are provided in Section 3.7.

Representative molecule-level case studies, showing how fusion corrects unimodal failures, are provided in the Appendix G.

## Discussion

### From proxy to practice: validating the HITL pathway

Our human validation study (Section 4.5) serves as the core validation for our central thesis that a scalable proxy-HITL system can transition to a truly collaborative HITL framework. The experiment demonstrates that the framework’s knowledge interface is fundamentally source-agnostic, a principle evidenced by the successful performance enhancement (AUROC increasing from 0.871 to 0.889). The fact that this gain was achieved by swapping LLM-generated insights with human-refined ones, critically without any retraining, confirms that the proxy is not merely a simulation but a functional placeholder in a system architected for genuine human expertise.

This finding elevates our contribution beyond a simple performance improvement. It establishes an empirical blueprint for a hybrid intelligence workflow where the scalability of LLMs can handle routine cases, while human chemists can be seamlessly integrated to resolve high-uncertainty challenges identified by the system. Therefore, while the current implementation operates as a proxy, this proof-of-concept study provides direct validation for the entire HITL pathway, bridging the gap between automated systems and indispensable expert intuition in drug discovery.

### Synergy and robustness of the proxy-HITL Design

The above analyses underscore two key findings. First, the robustness study shows that LLM-generated chemist knowledge is semantically stable even under stochastic decoding, alleviating concerns of variability and hallucination. Second, fusion consistently corrects modality-specific weaknesses: structure-only models may overlook high-level pharmacophoric reasoning, while knowledge-only models lack structural specificity. By integrating the two, MolProphecy achieves more reliable and interpretable predictions.

These results highlight the practical value of the proxy-HITL design. The framework not only provides measurable gains in predictive accuracy but also offers interpretable rationales that mirror chemists’ reasoning processes, thereby improving trustworthiness for downstream drug discovery tasks.

### Contributions and limitations

MolProphecy introduces a multi-modal framework that integrates chemist-inspired knowledge with graph-based molecular representations for property prediction. Unlike prior approaches that treat chemist expertise as auxiliary annotations, we consider it an independent modality, co-equal to molecular structure. This expertise, currently simulated by ChatGPT but designed to be replaceable by real chemists without retraining, enables alignment between human reasoning and data-driven modeling. Our framework achieves consistent performance gains across regression and classification tasks on nine benchmark datasets, while interpretability analyses (PCA, entropy, SHAP, and SME) reveal that fused representations are more expressive and informative than either modality alone.

Despite these advances, several limitations remain. First, the quality of LLM-generated insights depends on prompt design and molecular context. For rare scaffolds or atypical functional groups, generated reasoning may be noisy or incomplete. Although fusion mitigates much of this variability, prompt sensitivity remains a potential weakness. Second, our external validation is currently limited to a regression dataset (solubility). We chose this setting because regression provides a stricter test of generalization due to continuous property distributions, complementing the classification benchmarks already covered by MoleculeNet. Future work will extend external validation to classification datasets once sufficiently independent benchmarks become available. Third, our current study does not systematically characterize failure conditions where fusion may be less effective, such as tasks requiring highly localized stereochemical or scaffold-specific information. Addressing these conditions requires further methodological refinement. Fourth, although MolProphecy is described as a HITL framework, the present implementation more closely resembles a proxy-HITL design rather than a fully interactive HITL system. Chemist expertise is simulated by LLMs, and humans do not continuously intervene during training or inference. This static knowledge injection differs from the dynamic, closed-loop feedback that defines strict HITL systems. To partially bridge this gap, we conducted a small-scale validation on BACE challenge cases (Section 4.5), where medicinal chemists refined LLM-generated insights and achieved measurable improvements in predictive accuracy. This result demonstrates both the feasibility and value of selective human involvement. A fully interactive HITL workflow, where chemists dynamically adjust knowledge granularity and correct outputs in real time, remains an important direction for future work.

## Conclusion

This study presents MolProphecy, a multi-modal framework that combines chemist-inspired knowledge with molecular graph embeddings to improve molecular property prediction. MolProphecy demonstrate improved performance and enhanced interpretability across diverse benchmarks, particularly highlighting the robustness gained through symbolic–structural fusion. The implementation is publicly available at https://github.com/zhangruochi/MolProphecy. By framing chemist expertise as a modular, source-agnostic knowledge channel, MolProphecy establishes a pathway toward scalable proxy-HITL drug discovery. Future work will focus on adaptive prompting, efficiency improvements, and more interactive chemist–model collaboration, moving from proxy-HITL toward more dynamic, closed-loop HITL systems.

## CRediT authorship contribution statement

Jianping Zhao: Conceptualization, Supervision, Project administration, Writing – original draft preparation, Writing – review and editing. Qiong Zhou: Conceptualization, Methodology, Supervision, Writing – original draft preparation, Writing – review and editing. Tian Wang: Methodology, Software. Yusi Fan: Methodology, Investigation. Qian Yang: Data curation, Software. Li Jiao: Validation, Data curation. Chang Liu: Software, Formal analysis. Zhehao Guo: Visualization, Formal analysis. Qi Lu: Visualization, Validation. Fengfeng Zhou: Funding acquisition, Resources, Supervision, Writing – review and editing. Ruochi Zhang: Methodology, Writing – review and editing, Supervision, Project administration.

## Declaration of Competing Interest

The authors declare that they have no known competing financial interests or personal relationships that could have appeared to influence the work reported in this paper.

## Figures and Tables

**Table E.12:** Dataset splitting strategies for all MoleculeNet benchmarks used in this study.

**Dataset**	**Split Type**	**Ratio (Train/Val/Test)**	**Notes**
FreeSolv	Random	8:1:1	Regression (hydration free energy)
ESOL	Random	8:1:1	Regression (solubility)
Lipophilicity	Random	8:1:1	Regression (logP)
BBBP	Scaffold	8:1:1	Blood–brain barrier penetration
BACE	Scaffold	8:1:1	β-secretase inhibition
HIV	Scaffold	8:1:1	Antiviral activity
SIDER	Scaffold	8:1:1	27 adverse-reaction subtasks
ClinTox	Scaffold	8:1:1	Toxicity endpoints
Tox21	Scaffold	8:1:1	12 toxicity targets

**Table E.13:** Preprocessing and overlap removal for the independent solubility dataset.

**Stage**	**# Molecules**	**Notes**
Original Ulrich dataset	1291	Reported in Ulrich [41]
After overlap removal	949	342 duplicates discarded
After invalid SMILES removal	949	Final cleaned dataset

**Table F.14:** Re-implementation of regression baselines. Results are reported as RMSE (↓). Bold indicates the best performance.

**Method**	**FreeSolv**	**ESOL**	**Lipophilicity**
D-MPNN	1.89 ± 0.41	0.94 ± 0.15	0.73 ± 0.05
ChemBERTa	1.83 ± 0.25	0.875 ± 0.22	0.776 ± 0.14
MolPROP	1.63 ± 0.11	0.781 ± 0.13	0.741 ± 0.09
**MolProphecy (Ours)**	**0.796** ± **0.09**	**0.693** ± **0.006**	**0.657** ± **0.07**

**Table G.15:** Representative BACE molecules where the fusion model (MolProphecy-Full) correctly predicts the outcome, while at least one unimodal variant (MolProphecy-GIN or MolProphecy-Chem) fails. Correct predictions are highlighted in green and incorrect ones in red.

## Task-specific prediction descriptions

The following descriptions correspond to the {task-specific description} fields referenced in the prompt template. They define the prediction objectives for each benchmark dataset used in our experiments.

• FreeSolv: This task involves predicting the hydration free energy of small molecules in water, which reflects solvation behavior and plays a key role in drug absorption and distribution.
• BACE: A binary classification task for predicting the binding affinity of molecules with human beta-secretase 1 (BACE-1), a therapeutic target in Alzheimer’s disease.
• ClinTox: A toxicity classification task that aims to distinguish drugs approved by the FDA from those that have failed clinical trials due to toxicity.
• SIDER: This multi-label classification task predicts potential adverse drug reactions (ADRs) across 27 physiological systems. The full list of ADR categories is as follows:
  1. Hepatobiliary disorders
  2. Metabolism and nutrition disorders
  3. Product issues
  4. Eye disorders
  5. Investigations
  6. Musculoskeletal and connective tissue disorders
  7. Gastrointestinal disorders
  8. Social circumstances
  9. Immune system disorders
  10. Reproductive system and breast disorders
  11. Neoplasms (benign, malignant, and unspecified)
  12. General disorders and administration site conditions
  13. Endocrine disorders
  14. Surgical and medical procedures
  15. Vascular disorders
  16. Blood and lymphatic system disorders
  17. Skin and subcutaneous tissue disorders
  18. Congenital, familial, and genetic disorders
  19. Infections and infestations
  20. Respiratory, thoracic, and mediastinal disorders
  21. Psychiatric disorders
  22. Renal and urinary disorders
  23. Pregnancy, puerperium, and perinatal conditions
  24. Ear and labyrinth disorders
  25. Cardiac disorders
  26. Nervous system disorders
  27. Injury, poisoning, and procedural complications
• HIV: Binary classification task predicting compounds that inhibit HIV replication.
• BBBP: Binary classification task predicting blood–brain barrier penetration of small molecules.
• tox21: Toxicity prediction benchmark with 12 classification tasks, covering nuclear receptor signaling and stress response pathways.
• esol: Regression task predicting aqueous solubility (logS) for drug-like molecules.
• Lipophilicity: Regression task predicting octanol–water partition coefficient (logP), an indicator of lipophilicity.
• independent dataset: Regression task predicting aqueous solubility (logS) for drug-like molecules. The goal is to estimate experimental solubility (Sexp) from molecular structure (SMILES).

## Prompt for semantic similarity scoring

*Prompt.*
You are a professional chemist. Given two chemist-style molecular insights (A and B), evaluate how semantically similar they are in terms of described structural features, physicochemical properties, and reasoning. Use a scale from 1 to 5:

1. Completely unrelated content
2. Slightly related
3. Moderately similar
4. Highly similar
5. Nearly identical in meaning

Only output a single number (1–5) without explanation. Insight A: {text1}. Insight B: {text2}. Similarity score (1 to 5):

## Input features for molecular graph encoder

The Molecular Graph Encoder in MolProphecy follows the featurization scheme adapted from MolCLR [43]. Each molecule is represented as a graph G=(V,E), with atoms as nodes and bonds as edges. Atom and bond features are embedded as follows:

*Atom features.* Each atom is characterized by:

• Atom type: One-hot encoding over 118 possible elements plus one mask token (119 categories).
• Chirality: Three categories (unspecified, clockwise, counterclockwise).

*Bond features.* Each bond is represented by:

• Bond type: Five categories (single, double, triple, aromatic, dative), plus a self-loop edge type.
• Bond direction: Three categories (none, end-up-right, end-down-right), encoding stereochemistry.

During message passing, the final edge embedding is computed as the sum of bond-type and bond-direction embeddings, while self-loops are explicitly added with a designated bond type index.

## Prompt for consensus analysis judge

*Prompt.*
You are an expert medicinal chemist acting as a neutral arbiter. Your task is to review three different expert insights (Insight A, Insight B, Insight C) for the same molecule and determine if there is a **significant conflict** in their chemical reasoning. **INPUTS:**

• **Insight A (from ChatGPT):** {text1}
• **Insight B (from Qwen3):** {text2}
• **Insight C (from Gemini):** {text3}

**INSTRUCTIONS:** A **significant conflict** exists if there is a clear contradiction in any of the following key areas:

1. **Functional Group Identification:**e.g., one insight identifies a key functional group that another misses or misidentifies (e.g., ’amide’ vs. ’ester’).
2. **Physicochemical Property Assessment:**e.g., one insight describes the molecule as ’highly lipophilic’ while another calls it ’hydrophilic’.
3. **ADMET or Pharmacophore Heuristics:**e.g., one insight suggests a high potential for toxicity or a specific pharmacophore interaction, while the others make a contradictory claim or completely omit this critical point.

**Important:** Minor differences in wording, phrasing, or focus do **not** constitute a conflict as long as the underlying chemical reasoning is consistent. **OUTPUT FORMAT:** Based on your analysis, answer with a single word: **’Conflict’** or **’No Conflict’**. Do not provide any explanation or additional text. **Your decision:**

## Dataset details and splitting strategies

Additional details on dataset splitting and distribution characteristics are provided to complement the descriptions in the main text, we provide additional details on dataset splitting and distribution characteristics. Table E.12 summarizes the splitting strategies used across the nine MoleculeNet benchmarks, while Fig. E.10 visualizes their label or property distributions.

The independent solubility dataset introduced by Ulrich [41] originally contains 1291 curated molecules with experimental solubility values. To ensure independence from MoleculeNet, canonical SMILES from all MoleculeNet benchmarks were collected and compared against the Ulrich dataset. Overlap removal and validity checks resulted in a final set of 949 unique molecules used in the external validation experiments (Section ‘External validation on an independent dataset’). The preprocessing workflow and molecule counts at each stage are summarized in Table E.13.

## Additional evaluation

To complement the classification results in the main text, we also revisited the regression benchmarks (FreeSolv, ESOL, Lipophilicity). To ensure a fair comparison, we re-implemented key regression baselines (D-MPNN, ChemBERTa, and MolPROP) using their publicly available code under identical random split settings (8:1:1). While the original implementations sometimes used different data partitions or training configurations, we followed their commonly reported hyperparameters where available and applied them consistently across all datasets. As summarized in Table F.14, MolProphecy outperforms these baselines on all three regression datasets, demonstrating the robustness of our framework in predicting continuous-valued molecular properties.

## Case study insights

Building on the consistency analysis, we investigate the robustness of MolProphecy-Full in scenarios where unimodal reasoning fails. Specifically, we analyzed six representative BACE molecules (Table G.15). Each case was correctly predicted by MolProphecy-Full, while at least one unimodal MolProphecy-GIN or MolProphecy-Chem failed. These failures fall into three categories, reflecting different limitations in unimodal reasoning and demonstrating how multi-modal integration compensates for them.

The first type includes Molecules A and B, where both MolProphecy-GIN and MolProphecy-Chem failed. Their LLM-generated chemist insights focus on general physicochemical features without referencing target-specific pharmacophores. The insight for Molecule A states: ”The molecule has a TPSA value of 156.21, indicating good solubility. Structurally, it contains aromatic rings, an amide bond, a sulfonyl group, and a nitrile group. These functional groups suggest potential interactions with the target BACE-1 enzyme”. For Molecule B, the insight describes it as ”a medium-sized compound with a molecular weight of 415.429” with ”a low CLogP value of 0.256, making it relatively hydrophilic” and ”a sulfonamide group, an aniline moiety, and a tertiary amine” identified as potential pharmacophores. While chemically reasonable, these insights lack specific functional interpretations tied to BACE-1 inhibition. Without strong signals from either modality, both unimodal models failed. MolProphecy-Full, by contrast, successfully combines weak complementary cues from chemist knowledge and structural encoding to make the correct prediction.

The second failure mode includes Molecules C and D, where MolProphecy-GIN succeeded but MolProphecy-Chem failed. The chemist insights for these molecules reference relevant fragments but express them in overly general terms. For Molecule C, the insight mentions ”aromatic rings, amides, and hydrogen bond donor/acceptor groups”, noting that ”similar approved drugs may have structures with aromatic rings, amides, and potential hydrogen bond donor/acceptor groups”. However, it does not provide specific evidence of how these features contribute to BACE inhibition. The insight for Molecule D highlights ”a relatively high CLogP value of 4.089”, ”5 hydrogen bond acceptors and 1 donor” and an ”amide functional group” as potentially involved in protein binding interactions. These high-level descriptions may cause MolProphecy-Chem to overgeneralize across diverse compounds. In contrast, MolProphecy-GIN captures structural patterns specific to the task and provides the correct classification. MolProphecy-Full reconciles the symbolic cues from the chemist insight with molecular structure, leading to a reliable prediction.

The third category comprises Molecules E and F, where MolProphecy-Chem succeeded but MolProphecy-GIN failed. These molecules exhibit structurally rich motifs such as sulfonyl groups, fluorinated aromatics, and peptidomimetic backbones. Their chemist insights offer more detailed pharmacophoric interpretation. The insight for Molecule E highlights ”sulfonyl, primary amine, and tertiary amine groups that can participate in various types of interactions with the target enzyme”. For Molecule F, it describes ”a peptidomimetic compound containing a fused tricyclic ring system”, noting that it ”may exhibit good interactions with the target enzyme” and possesses ”moderate polar surface area” and ”unique pharmacophore features”. These insights capture interaction potential that the structural model alone may not encode. MolProphecy-Full leverages this chemist-derived interpretation to produce correct predictions even when the structural encoding is insufficient. Full SMILES strings and chemist-style insights for all six molecules are provided below.

These case studies reinforce our earlier quantitative observations and highlight that MolProphecy-Full corrects prediction errors arising from modality-specific limitations. Even when chemist-style insights are vague or only partially informative, their integration with molecular structure leads to more reliable predictions.

*Molecule A.* **SMILES:** S(=O)(=O)(N(c1cc(ccc1) C(=O) NC(Cc1ccccc1) C(O) C[NH2+]C(C(=O) NC1CCCCC1) C) c1ccc(cc1) C#N) C

**Insight:** The provided molecule is a large, complex molecule with a molecular weight of 632.807. It has 6 hydrogen bond acceptors, 4 hydrogen bond donors, and a high number of rotatable bonds (13). The molecule has a TPSA value of 156.21, indicating good solubility. Structurally, it contains aromatic rings, an amide bond, a sulfonyl group, and a nitrile group. These functional groups suggest potential interactions with the target BACE-1 enzyme. The molecule may have suitable physicochemical properties for binding to the enzyme based on its size, polarity, and functional groups.

*Molecule B.* **SMILES:** S1(=O) CC(Cc2cc(OC(COC) C(F)(F) F) c(N) c(F) c2) C(O) C([NH3+]) C1

**Insight:** The provided molecule is a medium-sized compound with a molecular weight of 415.429. It has a low CLogP value of 0.256, making it relatively hydrophilic. With 5 hydrogen bond acceptors and 3 donors, it exhibits moderate potential for interacting with target proteins. The molecule contains multiple rotatable bonds (RB = 6) which may impact its conformational flexibility. The TPSA value of 109.42 suggests a moderate surface area available for interactions. The presence of a sulfonamide group, an aniline moiety, and a tertiary amine highlights potential pharmacophores for binding to the active site of BACE-1.

*Molecule C.* **SMILES:** IC1 = CN = C(NC1 = O) C(NC1 = NC(Cc2c1ccc(Cl) c2)(C) C) Cc1ccccc1

**Insight:** The molecule has a molecular weight of 532.813, a CLogP of 4.683, 4 hydrogen bond acceptors, 2 hydrogen bond donors, and 4 rotatable bonds. It also has a TPSA of 70.14. The structural analysis indicates a complex and bulky structure with multiple aromatic rings and a pyrazinone core. Key functional groups include amide, amine, and aromatic rings. The molecule’s size and complexity suggest potential binding to a protein target like BACE-1. Stereochemistry and key fragments could play a role in determining the molecule’s binding affinity. Similar approved drugs may have structures with aromatic rings, amides, and potential hydrogen bond donor/acceptor groups.

*Molecule D.* **SMILES:** O(CCC) c1cc(ccc1)-c1ccc(cc1) C1CC1C = 1 N = C(N) N(C) C(=O) C = 1

**Insight:** The provided molecule is a medium-sized compound with a molecular weight of 375.472. It has a relatively high CLogP value of 4.089, indicating moderate lipophilicity. The molecule has 5 hydrogen bond acceptors and 1 hydrogen bond donor, suggesting potential interactions with protein targets. It contains a total of 6 rotatable bonds, which may affect its conformational flexibility. The TPSA value of 70.14 indicates moderate polar surface area. The presence of the amide functional group suggests potential involvement in protein binding interactions, making it a suitable candidate for the predictive task of identifying inhibitors of human beta-secretase 1 (BACE-1).

*Molecule E.* **SMILES:** S1(=O)(=O) CC(Cc2cc(-c3occc3) c(N) c(F) c2) C(O) C([NH2+]Cc2cc(ccc2) C(C)(C) C) C1

**Insight:** The provided molecule is a large peptide-like compound with a molecular weight of 501.644. It has a moderate hydrophobicity (CLogP: 3.046) and high hydrogen bond acceptor count (HBA: 5), indicating potential interactions with target proteins. The presence of multiple HBD (HBD: 3) groups suggests it may form hydrogen bonds with protein residues. The molecule is structurally complex with 6 rotatable bonds (RB: 6) and a relatively large polar surface area (TPSA: 110.14). The molecules contains sulfonyl, primary amine, and tertiary amine groups that can participate in various types of interactions with the target enzyme. Overall, the molecule possesses key features that may contribute to its potential as a BACE-1 inhibitor.

*Molecule F.* **SMILES:** O = C(c1cc2CN(CCC(=O) N(C) C3CCCCC3) C(=[NH+]c2cc1) N) c1ccccc1

**Insight:** The provided molecule is a peptidomimetic compound containing a fused tricyclic ring system. It has moderate molecular weight (419.549 g/mol) and low lipophilicity (CLogP: 1.941), indicating good drug-like properties. With 4 hydrogen bond acceptors and 2 donors, it may exhibit good interactions with the target enzyme. The molecule contains multiple rotatable bonds (RB: 6), suggesting flexibility in binding. The TPSA value of 80.61 Å^2^ indicates moderate polar surface area. The structure may potentially interact with the active site of BACE-1 due to its unique pharmacophore features.
